# Getting to the root of nodulation: how legumes and rhizobia use nitrate uptake to control symbiosis

**DOI:** 10.1093/plcell/koac065

**Published:** 2022-02-21

**Authors:** Lucas Frungillo

**Affiliations:** Assistant Features Editor, The Plant Cell, American Society of Plant Biologists, USA; School of Biological Sciences, Institute of Molecular Plant Sciences, University of Edinburgh, Edinburgh EH9 3BF, UK

Nitrogen (N) is an essential component of various biomolecules, such as amino acids and nucleic acids. A major source of N to land plants is the inorganic ion nitrate (NO_3_^−^). To uptake NO_3_^−^ from the soil, plants rely on the activity of sophisticated NO_3_^−^ transporter (NRT) systems in roots (Vidal et al., 2020). However, under limiting N conditions, some plants evolved the remarkable ability to acquire atmospheric N. Legumes, for example, establish symbiotic relationships with N-fixing soil bacteria from the genus *Rhizobia* to form highly specialized structures, called nodules, in their roots (reviewed in Roy et al., 2020). In nodules, rhizobia fix atmospheric N at the expense of photosynthetic products provided by plants. Although beneficial under N limiting conditions, nodulation is energetically expensive to plants and, thus, tightly regulated. Members of the NLP family of transcriptional factors suppress nodulation in legumes by orchestrating gene expression in response to N supply. Despite recent advances in understanding the genetic cascades required for nodulation, the molecular mechanisms underlying the switch between N acquisition strategies remain unclear. In this issue of *The Plant Cell*, **Misawa and colleagues (****Misawa et al., 2022****)** uncover a transcriptional cascade involving NLPs and an NRT controlling the interplay between NO_3_^−^ uptake and nodulation in the legume *Lotus japonicus*. Their findings expand our understanding of how plants control nodulation and may substantiate efforts on the development of sustainable strategies to improve plant performance.

To investigate the genetic basis of switching between N acquisition strategies in legumes, the authors took advantage of an *L. japonicus* mutant library previously assayed for crosstalk between nitrate and nodulation (Nishida et al., 2018). Genome-resequencing and DNA-based phylogenetic analysis of independent *L. japonicus* mutants with nitrate-insensitive nodules (see Figure) revealed missense mutations in a single gene encoding a protein with similarities to those in the *Arabidopsis thaliana* NRT2 gene family. In Arabidopsis, the *NRT2* gene family is composed of high-affinity nitrate transporters that mediate nitrate uptake under low N availability. Accordingly, phenotypic analysis revealed that *Ljnrt2.1* mutants display reduced nitrate uptake capacity and biomass accumulation compared with wild-type plants. Because mutation of LjNLP1 and LjNLP4 transcription factors results in a similar nodulation phenotype as the newly identified *Ljnrt2.1* (Nishida et al., 2021), the authors investigated if LjNLP1, LjNLP4, and LjNRT2.1 formed a signalling module. Interestingly, transcriptional analysis and transactivation assays indicated that NLP1 positively regulates *NRT2.1* expression in *L. japonicus*. Additionally, LjNLP4 nuclear localization and downstream gene expression in response to nitrate is impaired in *Ljnlp1* and *Ljnrt2.1* mutant plants, suggesting that nitrate uptake and downstream transcriptional reprogramming control nodulation. To further establish that NO_3_^−^ transport underpins the switch between N acquisition strategies, the authors analyzed the impact of the transcription factor LjNIN, a positive regulator of nodulation, on the newly identified transcriptional module. Co-expression and electrophoretic mobility shift assays showed that LjNIN counteracts LjNLP1 by competitively binding to cis-elements of the *NRT2.1* promoter. Together with transcriptional analyses presented, these data suggest that rhizobia utilize the NLP1/4-NRT2.1 signaling module to promote nodulation. The authors then propose a model in which the switch between different N acquisition strategies is mediated by an interplay between transcription factors and nitrate uptake that is regulated by host and rhizobia.

**Figure:**
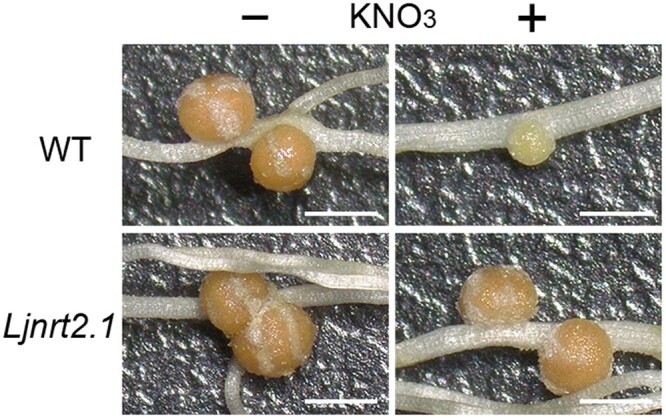
Nitrate transporter NRT2.1 controls nodulation in *L. japonicus*. Nodulation phenotypes of wild-type (WT) and *Ljnrt2.1* mutant in the presence or not of nitrate (KNO_3_). Scale bars = 1 mm. Adapted from Misawa et al. (2022), Figure 3.

Engineering N-fixing nodule symbiosis into non-legume crops is an attractive strategy to achieve sustainable agricultural production. **Misawa and colleagues’ (****Misawa et al., 2022****)** work uncovers a regulatory network that opens avenues for the identification of key players in the process of nodulation.
